# Psychometric Evaluation of the Bilingual Russian–Kazakh Problem Gambling Severity Index: Item-Level Response Patterns and Behavioral Factors Associated with Gambling-Related Harm

**DOI:** 10.3390/bs16091631

**Published:** 2026-09-12

**Authors:** Olga Tapalova, Nurgul Suyundykova, Nadezhda Zhiyenbayeva, Yermek Buribayev, Sultan Tapalov

**Affiliations:** 1 Department of General and Applied Psychology, Faculty of Pedagogy and Psychology, Abai Kazakh National Pedagogical University, Almaty 050010, Kazakhstan; o.tapalova@abaiuniversity.edu.kz; 2 Department of Special Pedagogy, Abai Kazakh National Pedagogical University, Almaty 050010, Kazakhstan; n.zhienbaeva@abaiuniversity.edu.kz; 3 Department of Law, Zhetysu University named after Ilyas Zhansugurov, Taldykorgan 040009, Kazakhstan; e.buribayev@zu.edu.kz; 4 Budgeting and Economic Analysis Department, Kazakh-Chinese Pipeline, Almaty 050008, Kazakhstan; s.tapalov@kcp.kz

**Keywords:** Kazakhstan, problem gambling severity, gambling, behavioral disorder

## Abstract

Population-level evidence on gambling-related risk remains limited in Central Asia, and no validated bilingual screening instrument has been available in Kazakhstan. This study evaluated the psychometric performance of the bilingual Kazakh–Russian Problem Gambling Severity Index (PGSI) and examined the distribution and correlates of PGSI-defined gambling severity in a multi-regional sample. A total of 1015 adults aged 18–60 years were recruited across all 17 regions and three cities of republican significance. Among the 474 respondents who directly completed all nine PGSI items, internal consistency was good (Cronbach’s α = 0.846), and the first principal component accounted for 45.9% of the variance. Of these respondents, 275 (58.0%) met the conventional PGSI ≥ 8 screening threshold. The same 275 respondents constituted 27.1% of the overall survey sample (N = 1015). These percentages reflect different analytic denominators and are interpreted as sample-specific rather than as estimates of national prevalence. Item-level patterns indicated that elevated scores predominantly reflected cumulative moderate-frequency endorsements across multiple domains. In adjusted analyses, gambling expenditure and frequency showed the strongest associations with PGSI ≥ 8 status. Continuous-score analyses yielded a broadly consistent pattern, indicating that the principal behavioral associations were not solely attributable to dichotomization at the screening threshold. These findings provide initial psychometric evidence for the bilingual PGSI in Kazakhstan and support continued psychometric investigation before its use in epidemiological research or population-level screening.

## 1. Introduction

Gambling disorder is recognized in both the Diagnostic and Statistical Manual of Mental Disorders (DSM-5) and the International Classification of Diseases (ICD-11) as a behavioral addiction characterized by persistent gambling despite harmful consequences and impaired control over gambling behavior. Beyond its clinical manifestations, gambling-related harm encompasses a broader continuum of adverse financial, psychological, interpersonal, and occupational consequences that affect not only individuals but also their families and communities (Kristensen et al., 2024; Tulloch et al., 2023). Contemporary public health research increasingly emphasizes the identification of gambling-related harm across the full spectrum of risk rather than focusing exclusively on clinically diagnosed gambling disorder (Price et al., 2021).

International epidemiological studies indicate substantial variation in gambling-related harm across populations. Recent reviews estimate the annual prevalence of gambling disorder among adults to range between approximately 1.1% and 3.5%, while broader categories of problem gambling have been reported in 2.3–12.6% of the adult population depending on assessment methods and national context (Moreira et al., 2024; Hodgins et al., 2022). These differences reflect not only genuine variation in gambling behaviour but also methodological diversity, differences in gambling environments, and the availability of validated screening instruments. Therefore, reliable population-based screening is essential for estimating gambling-related harm, monitoring trends, and informing prevention strategies.

The rapid expansion of online gambling, sports betting, and digital gambling platforms has further increased the importance of population surveillance. Digital gambling environments provide continuous access, reduce traditional barriers to participation, and have been associated with increased gambling involvement and harm (Watanapongvanich et al., 2020). At the same time, gambling-related problems frequently remain under-recognized because of stigma, delayed help-seeking, and limited routine screening within healthcare and social services (Quigley, 2022; Jääskeläinen & Kuusisto, 2025; Jones et al., 2025). Consequently, prevalence estimates based solely on clinical diagnoses are likely to underestimate the actual burden of gambling-related harm.

Beyond its use in population research, increasing attention has been directed toward the identification of gambling-related harm within healthcare and community settings. Recent evidence suggests that brief gambling screening may facilitate early identification and referral in primary-care and mental-health contexts, while positive screening results require further assessment rather than being interpreted as diagnostic classifications (Reid et al., 2024; Roberts et al., 2025). From a broader public health perspective, systematic identification of gambling-related harm may also contribute to prevention and surveillance strategies (Clune et al., 2024).

Kazakhstan represents one of the least studied gambling environments in Central Asia. Despite the rapid development of legal gambling opportunities and online betting markets, large-scale multi-regional epidemiological evidence remains unavailable. Official health statistics report only a small number of clinically diagnosed gambling disorder cases despite a population exceeding 20 million, suggesting substantial under-identification rather than low occurrence. Moreover, no standardized national screening system has been implemented to estimate gambling-related harm in the general population, limiting the evidence available for prevention planning and public health policy.

A further limitation concerns the absence of a validated bilingual screening instrument suitable for Kazakhstan’s Russian- and Kazakh-speaking population. The Problem Gambling Severity Index (PGSI) is among the most widely used instruments for measuring gambling-related harm in community samples (Ferris & Wynne, 2001). Unlike diagnostic interviews, the PGSI conceptualizes gambling-related harm as a continuum and has demonstrated good reliability and validity across diverse cultural settings. Nevertheless, its psychometric performance has not previously been evaluated in Kazakhstan, and no evidence exists regarding the response patterns of individual PGSI items within this population.

The present study addresses these gaps by providing the first psychometric evaluation of the bilingual Russian–Kazakh PGSI in a large multi-regional sample of adults in Kazakhstan. In addition to examining the reliability and construct validity of the instrument, this study describes item-level response patterns, estimates the national distribution of gambling-related harm, and identifies behavioral and sociodemographic factors independently associated with elevated gambling-related risk. Collectively, these findings provide an empirical foundation for future epidemiological surveillance, cross-cultural research, and evidence-informed prevention strategies targeting gambling-related harm in Kazakhstan.

Accordingly, the present study aimed to evaluate the psychometric performance of the bilingual Kazakh–Russian PGSI and to examine the distribution and correlates of PGSI-defined gambling risk in a large multi-regional adult sample from Kazakhstan. Based on the established psychometric properties of the PGSI, we hypothesized that: (H1) the bilingual PGSI would demonstrate satisfactory internal consistency and a predominantly unidimensional factor structure; (H2) the underlying construct measured by the PGSI would show comparable measurement properties across the Kazakh- and Russian-language versions; and (H3) greater gambling involvement, particularly more frequent gambling and a greater proportion of income spent on gambling, would be associated with higher PGSI scores. Given the absence of prior population-level PGSI data from Kazakhstan and the sampling design of the present study, no a priori expectation was specified regarding the proportion of participants falling within individual PGSI screening categories. Sociodemographic associations, the distribution of PGSI scores and screening categories, and item-level response patterns were therefore examined as exploratory objectives.

## 2. Literature Review

Gambling-related harm is now viewed as a multidimensional continuum encompassing financial, psychological, interpersonal, occupational, and health-related consequences that emerge across varying levels of gambling involvement rather than solely among individuals meeting diagnostic criteria (Langham et al., 2016; Browne et al., 2017). This public health perspective has fundamentally altered approaches to gambling research and prevention. Rather than considering gambling disorder exclusively as an individual psychiatric condition, researchers increasingly emphasize the cumulative burden of gambling-related harm across populations. Browne et al. (2017) demonstrated that although severe gambling disorder accounts for considerable individual suffering, a substantial proportion of gambling-related harm occurs among individuals experiencing low- and moderate-risk gambling because these groups represent a much larger proportion of the population. Consequently, public health frameworks advocate shifting attention from treatment alone toward prevention, early identification, and systematic surveillance (Kristensen et al., 2024).

International epidemiological evidence demonstrates considerable variability in gambling-related harm across countries. Recent systematic reviews estimate the annual prevalence of gambling disorder among adults to range between approximately 1.1% and 3.5% (Moreira et al., 2024), whereas broader categories of problem gambling have been reported in 2.3–12.6% of adult populations depending on assessment methodology, gambling availability, and national context (Hodgins et al., 2022). Such variability reflects not only genuine differences in gambling behavior but also differences in legislative frameworks, gambling availability, cultural attitudes, and the psychometric performance of screening instruments. This highlights the need for culturally appropriate and psychometrically validated measures capable of identifying gambling-related harm across the full spectrum of severity rather than solely among clinically diagnosed cases.

Beyond prevalence estimates, gambling-related harm constitutes a significant public health concern owing to its consistent association with psychiatric comorbidity, financial hardship, social dysfunction, reduced quality of life, and increased mortality (Karlsson & Håkansson, 2018). Moreover, the rapid expansion of online gambling, sports betting, esports wagering, and mobile gambling platforms has intensified these concerns by increasing both the accessibility and frequency of gambling opportunities (Granero et al., 2020; López-González et al., 2018a; Moreira et al., 2023; Watanapongvanich et al., 2020).

Despite the growing recognition of gambling-related harm, early identification remains challenging because gambling often develops within sociocultural contexts that discourage disclosure. Stigma is among the most important barriers, as gambling problems are frequently perceived as signs of personal irresponsibility or moral failure rather than manifestations of an addictive disorder, thereby reducing help-seeking behavior (Hing et al., 2016). Self-stigma further reinforces these barriers through feelings of shame, guilt, and anticipated social rejection, resulting in concealment of gambling behaviors until financial, interpersonal, or psychological consequences become severe (Quigley, 2022). These processes contribute not only to delayed treatment seeking but also to systematic underestimation of gambling-related harm within epidemiological studies that rely primarily on healthcare utilization or clinical diagnosis.

The normalization of gambling within contemporary leisure environments further complicates early recognition. Gambling has become increasingly embedded in sports, online gaming, esports, and digital media, blurring the distinction between recreational participation and harmful involvement (Watanapongvanich et al., 2020). As a result, gambling is often perceived as a socially acceptable activity rather than a potential health concern, particularly in online environments where participation occurs privately and is less visible than in traditional gambling venues.

Sociocultural barriers are further reinforced by institutional limitations. Jääskeläinen and Kuusisto (2025) argue that gambling-related problems remain insufficiently addressed within routine healthcare and social services because standardized screening procedures are rarely implemented outside specialized addiction settings. Similarly, Jones et al. (2025) emphasized that delayed recognition frequently reflects systemic deficiencies in professional education and assessment practices rather than the absence of clinically significant symptoms. Consequently, gambling-related harm represents not only an individual behavioral phenomenon but also a condition whose visibility is shaped by social attitudes, institutional practices, and the availability of sensitive assessment strategies.

These observations have important implications for gambling assessment. Contemporary research increasingly recognizes that delayed identification reflects not only stigma and limited access to care but also conceptual and psychometric limitations of existing assessment approaches. Gambling-related harm develops along a continuum of behavioral, cognitive, emotional, and motivational processes rather than as a discrete condition (Molander et al., 2021). However, many diagnostic frameworks continue to rely on categorical thresholds, reducing sensitivity to early or subclinical manifestations of gambling-related risk. Although categorical diagnoses remain essential for clinical decision-making, they are less effective in identifying emerging patterns of harm before diagnostic criteria are met, thereby limiting opportunities for early intervention.

The multidimensional nature of gambling-related harm is reflected in the psychological mechanisms underlying gambling persistence. Beyond monetary reinforcement, gambling behavior is sustained by cognitive distortions, emotional regulation, motivational processes, and reward expectations, including loss chasing, attentional preoccupation, illusion of control, and distorted outcome expectancies (Hing & Russell, 2017; Molander et al., 2021). Experimental evidence further demonstrates that structural characteristics of gambling products, such as near-miss effects and intermittent reinforcement, promote continued gambling despite objective losses (Çakıcı et al., 2021). Consequently, psychological vulnerability may emerge before behavioral indicators, including gambling frequency or expenditure, reach diagnostic thresholds.

These findings indicate that behavioral indicators alone provide an incomplete representation of gambling-related harm. Emotional vulnerability and cognitive distortions have been shown to improve the identification of gambling-related problems beyond behavioral measures (Nigro et al., 2021), supporting recommendations to integrate behavioral, cognitive, emotional, and motivational dimensions into contemporary screening approaches (Jääskeläinen & Kuusisto, 2025; Jones et al., 2025). The limitations of conventional assessment are further highlighted by emerging gambling-related activities—including speculative investing, esports betting, skin gambling, and loot boxes—which share important psychological characteristics with conventional gambling disorder despite frequently remaining outside traditional diagnostic classifications (Lee et al., 2023; Moreau et al., 2016). Collectively, this evidence suggests that multidimensional screening instruments are better suited than categorical diagnostic thresholds to identify gambling-related harm across its full continuum.

Conceptual limitations in gambling assessment are further compounded by institutional factors affecting diagnostic capacity. Gambling disorder remains under-recognized because routine screening is rarely incorporated into healthcare services, while limited professional training contributes to inconsistent assessment and delayed recognition (Jones et al., 2025). As a result, gambling-related problems are often attributed to other psychosocial or psychiatric conditions without systematic evaluation of gambling behavior. Diagnostic complexity is further increased by high rates of psychiatric comorbidity, particularly with substance use, mood, and personality disorders, which increase the risk of diagnostic overshadowing (Mundt & Baranyi, 2020; Nigro et al., 2021). Similar patterns have been reported in psychiatric and forensic settings, where gambling behavior is infrequently assessed despite its contribution to functional impairment, offending behavior, and relapse risk (Corbeil et al., 2023; Hing et al., 2015). Together, these findings suggest that under-detection reflects not only limitations of screening instruments but also broader institutional barriers to routine identification of gambling-related harm.

These international findings are particularly relevant for Kazakhstan, where gambling research remains limited despite the rapid expansion of legal gambling, online betting, and digital gambling platforms. Between 1990 and 2020, no peer-reviewed studies authored by researchers affiliated with Kazakhstani institutions and indexed in major international databases examined gambling addiction as a primary research focus. Existing studies have largely addressed legal regulation or public policy rather than behavioral epidemiology or clinical assessment. Preliminary research has reported cognitive distortions and financial overextension among gambling participants (Prilutskaya & Kuliev, 2016), emphasized gambling as an emerging public health issue (Y. Buribayev & Khamzina, 2025), explored psychological correlates of gambling behavior (Y. A. Buribayev et al., 2025), and examined legislative and criminological aspects of gambling expansion (Berdaliyeva et al., 2023). However, no large multi-regional study has assessed gambling-related harm using internationally recognized screening instruments, and no psychometric validation of a bilingual gambling screening instrument has been conducted in Kazakhstan. In this context, standardized screening instruments provide an essential methodological foundation for population-based research. Although structured diagnostic interviews remain the clinical reference standard, screening instruments enable efficient identification of varying levels of gambling-related risk and facilitate comparisons across populations, particularly in countries where routine clinical surveillance and epidemiological evidence remain limited.

Among available screening instruments, the Problem Gambling Severity Index (PGSI) is one of the most extensively validated measures of gambling-related harm in community samples (Ferris & Wynne, 2001). Unlike diagnostic interviews, the PGSI conceptualizes gambling severity as a continuum, consistent with contemporary public health approaches emphasizing early identification of harmful gambling (Browne et al., 2017). Psychometric studies have demonstrated good reliability and validity across diverse cultural settings, with translated versions showing comparable factorial structures and psychometric performance (Currie et al., 2013; Casu et al., 2023; Molander et al., 2021; Caler et al., 2017; Loo et al., 2011). Recent evidence further highlights the importance of rigorous cross-cultural adaptation, particularly in multilingual populations (Bastiani et al., 2025; Chinawa et al., 2023). These characteristics make the PGSI particularly suitable for Kazakhstan, where no validated bilingual gambling screening instruments have previously been available.

The PGSI was selected based on its conceptual and psychometric suitability for population-based research. Unlike instruments primarily intended for clinical diagnosis, the PGSI was developed to assess problem-gambling severity in general-population samples and operationalizes gambling problems along a continuum of severity (Ferris & Wynne, 2001; Currie et al., 2013). Subsequent psychometric research has supported its unidimensional structure and measurement properties using confirmatory factor analysis and Rasch modelling (Miller et al., 2013). Of particular relevance to the present bilingual study, the PGSI has also undergone linguistic and cultural adaptation across diverse populations, with studies reporting satisfactory psychometric properties for Chinese (Loo et al., 2011), Spanish (López-González et al., 2018b), and Persian versions (Griffiths & Nazari, 2021). Taken together, its population-oriented design, continuous conceptualization of problem-gambling severity, established psychometric properties, and accumulated evidence from cross-cultural adaptations provided a strong methodological basis for evaluating the PGSI within Kazakhstan’s bilingual Kazakh–Russian context.

In the present study, PGSI-defined gambling risk and gambling-related harm are treated as related but conceptually distinct constructs. The PGSI was developed as a population-based measure of the severity of gambling problems and provides a dimensional index of gambling-related risk rather than a clinical diagnosis (Currie et al., 2013; Miller et al., 2013). By contrast, gambling-related harm refers to the broader range of adverse financial, psychological, interpersonal, occupational, health, and social consequences attributable to gambling, which may occur across different levels of gambling involvement and are not confined to individuals meeting criteria for gambling disorder or the highest PGSI category (Langham et al., 2016; Browne & Rockloff, 2017). Accordingly, the conventional PGSI ≥ 8 threshold is interpreted in this study as a high-severity screening classification within the PGSI framework, rather than as a clinical diagnosis of gambling disorder or a direct measure of the presence or prevalence of gambling-related harm. Importantly, this threshold should not be regarded as a socially defining boundary; rather, it is intended to identify individuals who may benefit from further assessment, support, or preventive interventions. Its use should therefore facilitate access to appropriate care and prevention rather than contribute to labeling, stigma, or discrimination. Throughout the manuscript, PGSI scores and categories are therefore described in terms of PGSI severity or screening-defined gambling risk, whereas the term gambling-related harm is reserved for the broader spectrum of adverse consequences associated with gambling.

The increasing prevalence of online betting, mobile gambling, esports wagering, and other digital gambling formats further reinforces the need for screening instruments capable of detecting gambling-related harm across a continuum of severity (Granero et al., 2020; López-González et al., 2018a; Moreira et al., 2023). Despite extensive international application of the PGSI, no psychometric evaluation of a bilingual Russian–Kazakh version has been conducted, and no large-scale multi-regional study has assessed gambling-related harm in Kazakhstan using a standardized screening instrument. Moreover, little is known about item-level response patterns or the behavioral factors associated with elevated gambling-related risk in the Kazakhstani population. Accordingly, the present study aimed to evaluate the psychometric properties of the bilingual Russian–Kazakh PGSI, examine item-level response patterns, characterize the distribution of PGSI-defined gambling risk in a large multi-regional sample, and identify behavioral and sociodemographic factors associated with elevated gambling-related risk.

## 3. Methods

### 3.1. Study Design

This study employed a cross-sectional multi-regional survey to assess Problem Gambling Severity Index (PGSI) defined gambling risk among adults in Kazakhstan. The primary objective was to estimate the distribution of screening-defined gambling risk in a large multi-regional sample while evaluating the psychometric performance of the bilingual (Kazakh–Russian) version of the PGSI as a population-level screening instrument. A secondary objective was to identify demographic, socioeconomic, and gambling-related characteristics associated with elevated gambling-related risk.

The study was designed within a public health surveillance framework, recognizing that standardized screening instruments provide epidemiological evidence of the distribution of gambling-related harm rather than clinical diagnoses. Accordingly, the findings are interpreted as estimates of screening-defined gambling risk and should not be considered equivalent to the prevalence of clinically diagnosed gambling disorder. The study followed the Strengthening the Reporting of Observational Studies in Epidemiology (STROBE) recommendations for reporting cross-sectional research whenever applicable.

### 3.2. Measurement Instrument

Gambling-related harm was assessed using the Problem Gambling Severity Index (PGSI), a nine-item screening instrument developed as part of the Canadian Problem Gambling Index (Ferris & Wynne, 2001). The PGSI measures behavioral and psychosocial indicators of gambling-related harm, including betting beyond financial means, chasing losses, borrowing money to gamble, perceived loss of control, and gambling-related guilt. Each item is rated on a four-point Likert scale ranging from 0 (“Never”) to 3 (“Almost always”), producing a total score between 0 and 27. Consistent with international recommendations, total scores were categorized as no-risk (0), low-risk (1–2), moderate-risk (3–7), and problem gambling (≥8). Because the PGSI conceptualizes gambling-related harm along a continuum rather than as a dichotomous disorder, it is particularly suitable for population-based screening and detecting emerging or subclinical patterns of harm (James et al., 2016; Nower et al., 2013).

### 3.3. Translation and Cross-Cultural Adaptation

The Kazakh and Russian versions of the PGSI were developed following internationally accepted guidelines for the cross-cultural adaptation of patient-reported outcome measures (Beaton et al., 2000; Wild et al., 2005). The adaptation process included two independent forward translations, synthesis of the translated versions, two independent blind back-translations, and review by an expert committee to ensure semantic, conceptual, and cultural equivalence. Particular attention was paid to culturally sensitive concepts, including gambling terminology, financial affordability, and household-related expressions, to maximize conceptual equivalence across both language versions. Cognitive interviews with 25 respondents for each language version were conducted to evaluate clarity, comprehensibility, and cultural relevance. Feedback obtained during pilot testing informed minor wording refinements before implementation in the national survey.

### 3.4. Participants and Sampling

The study included 1015 participants, comprising 551 men (54.3%) and 464 women (45.7%) (Table 1). The age and occupational composition of the sample should be considered when interpreting the observed distribution of PGSI scores. Participants aged 18–35 constituted approximately 60% of the sample, and students represented the largest occupational group (20.2%). This demographic concentration is relevant because younger adults have consistently been identified as a group with greater vulnerability to gambling problems. A recent systematic review and meta-analysis across 18 countries found that younger adults had a significantly higher likelihood of problem gambling than middle-aged adults, although the magnitude of age-related differences varied across jurisdictions (Dellosa & Browne, 2024). University students have likewise been identified as a population in which gambling problems occur at comparatively elevated levels, with a large meta-analysis documenting substantial rates of both problem and probable pathological gambling across international student samples (Nowak, 2018). The overrepresentation of younger adults and students in the present sample may have contributed to an upward shift in the observed PGSI distribution, including the relatively high proportion of those meeting the PGSI ≥ 8 screening threshold. However, this demographic composition should be regarded as a potential source of sampling-related influence rather than as a causal explanation for the observed PGSI distribution. Accordingly, these findings are interpreted as sample-specific and should not be extrapolated to the age and occupational structure of the adult population of Kazakhstan.

The age distribution was relatively balanced across the three younger age groups. Participants aged 22–35 years represented the largest proportion of the sample (30.1%), followed by those aged 18–21 years (29.8%) and 36–45 years (29.7%), whereas respondents aged 46–60 years accounted for 10.4% of the study population.

Regarding marital status, 49.1% of participants were married and 42.8% were single. Divorced respondents constituted 5.1%, widowed respondents 0.9%, and participants reporting other marital statuses 2.2%.

Students represented the largest occupational group (20.2%), followed by respondents employed in trade and services (16.3%), industry and construction (10.8%), and education and science (10.0%). Participants working in business and management accounted for 7.4%, those employed in transport and logistics for 6.5%, while 6.3% of respondents were unemployed. The remaining 22.5% were distributed across other occupational categories.

For full-sample PGSI scoring, responses corresponding to no endorsement of a PGSI item were coded as “Never” (0), consistent with the scoring procedure used to derive the item-level response distributions and PGSI severity categories. Respondents with gambling involvement (n = 474) had directly observed responses across all nine PGSI items. Structural non-applicability was distinguished from item-level missingness, and no imputation of genuinely missing PGSI responses was performed.

The overall survey sample comprised 1015 participants. The questionnaire used a prespecified branching structure based on past-year gambling participation. Respondents entering the gambling-involvement branch completed all nine PGSI items (n = 474), whereas respondents routed away from this branch had structurally non-applicable PGSI items rather than directly observed item responses. Accordingly, primary item-level psychometric analyses in the present revision were restricted to the 474 directly assessed respondents.

#### 3.4.1. Data Preparation

Of the 1015 participants, 527 completed the Russian-language version and 488 completed the Kazakh-language version. Recruitment was conducted using a mixed online–offline approach within all 17 regions and the three cities of republican significance. Recruitment in both channels was conducted within a quota-based fieldwork framework, with geographic, sex, and age characteristics used to guide sample composition. Online and offline recruitment served as complementary recruitment channels rather than separate sampling strata. Questionnaire administration and data entry were standardized using the Simple Forms digital platform. Geographic units were used to ensure broad territorial coverage rather than as primary sampling units within a formal multistage cluster-sampling design. Because geographic units were used to structure territorial coverage rather than as primary sampling clusters within a formal multistage design, conventional model-based standard errors were used in the logistic regression analyses; cluster-adjusted standard errors were not applied.

#### 3.4.2. Data Cleaning

Of the 1078 questionnaires initially collected, 63 (5.8%) were excluded due to incomplete responses, duplicate records, or failure to meet data-quality and logical-consistency criteria, resulting in a final analytic sample of 1015 participants. Missing data were assessed at the item and variable levels. No imputation of genuinely missing PGSI item responses was performed. Structural non-applicability was distinguished from item-level missingness. Primary item-level psychometric analyses were conducted among the 474 respondents with directly observed responses across all nine PGSI items. The previously reported full-sample structural-zero representation was retained only for descriptive and sensitivity comparisons.

### 3.5. Quantitative Procedures and Statistical Analysis

Data were collected using a structured questionnaire administered in Kazakh and Russian. The questionnaire included three domains: sociodemographic characteristics, gambling behavior, and gambling-related harm assessed using the nine-item Problem Gambling Severity Index (PGSI). Additional items assessed gambling frequency, duration, preferred gambling modalities, gambling expenditure, perceived interpersonal consequences, attempts to reduce gambling, and exposure to gambling advertising.

Data quality was evaluated by examining missing values, duplicate records, logical consistency, questionnaire completeness, and denominator accuracy. PGSI scores were calculated according to the original scoring guidelines and classified into four categories: no-risk (0), low-risk (1–2), moderate-risk (3–7), and problem gambling (≥8).

Continuous variables are presented as means and standard deviations or medians and interquartile ranges, whereas categorical variables are reported as frequencies and percentages. Individual PGSI item-response distributions were also examined.

Because of the high proportion of PGSI ≥ 8 classifications among directly assessed respondents, PGSI scores were independently reconstructed from the original nine item-level variables using the standard 0–3 scoring procedure. Exploratory analyses examined PGSI ≥ 8 classification by age, student status, broad geographic grouping, and level of past-year gambling involvement.

Primary item-level psychometric analyses were conducted among respondents with directly observed responses to all nine PGSI items (n = 474). Internal consistency was evaluated using Cronbach’s alpha, corrected item–total correlations, and alpha-if-item-deleted estimates. Sampling adequacy and factorability were assessed using the Kaiser–Meyer–Olkin (KMO) measure and Bartlett’s test of sphericity. Principal component analysis (PCA) was used as an exploratory data-reduction procedure and was not interpreted as a latent-factor model.

Confirmatory factor analysis (CFA) was repeated in the directly assessed PGSI subgroup (n = 474). Because the nine PGSI items are four-category ordered indicators, the analysis was based on polychoric correlations using a diagonally weighted least-squares approach. Model fit was evaluated using χ^2^, the comparative fit index (CFI), Tucker–Lewis index (TLI), root mean square error of approximation (RMSEA) with its 95% confidence interval, and standardized root mean square residual (SRMR). Standardized factor loadings were examined at the item level.

Cross-language psychometric performance was examined in the directly assessed subgroup, comprising Russian-language (n = 255) and Kazakh-language (n = 219) respondents. Language-specific internal consistency was first evaluated using Cronbach’s alpha. Multi-group ordinal CFA was then conducted to examine cross-language measurement invariance. Configural, loading-invariance, and threshold-invariance models were compared sequentially, with changes in CFI (ΔCFI) and RMSEA (ΔRMSEA) considered alongside overall model fit.

Associations between PGSI categories and participant characteristics were examined using Pearson’s chi-square tests and Cramér’s V. Effect sizes for categorical associations were expressed as Cramér’s V, with 95% confidence intervals estimated using nonparametric bootstrap resampling (3000 resamples). For significant omnibus associations involving multicategory variables, adjusted standardized residuals were examined to identify categories contributing to the overall association.

Gambling frequency was treated as an ordinal variable with five categories (never, less than once a month, 1–3 times per month, 1–2 times per week, and more than twice per week), and the share of monthly income spent on gambling comprised four ordered categories (<10%, 10–25%, 26–50%, and >50%). Higher values represented greater gambling frequency and a larger share of income spent, respectively.

Multivariable binary logistic regression was performed to examine factors independently associated with PGSI ≥ 8 screening status, with results reported as adjusted odds ratios (aORs) and 95% confidence intervals. Sex, age group, share of monthly income spent on gambling, gambling frequency, and duration of gambling involvement were included as predictors. The probability of meeting the PGSI ≥ 8 screening threshold was modeled using the logistic function:

P(Y=1|X)=exp(β0+∑i=1βikXi)1+exp(β0+∑i=1βikXi)

where Y = 1 denotes PGSI ≥ 8, X_i_ represents the predictor variables, and β_i_ represents the corresponding regression coefficients.

Model performance was evaluated using Nagelkerke’s R^2^, the Hosmer–Lemeshow goodness-of-fit test, and the area under the receiver operating characteristic curve (AUC). To assess the robustness of the regression findings, gambling frequency and the share of monthly income spent on gambling were additionally examined as categorical predictors rather than assuming a linear ordinal trend. An additional multiple linear regression treated total PGSI score as a continuous outcome to assess whether the observed associations depended on dichotomization at the PGSI ≥ 8 threshold.

Sensitivity analyses compared psychometric estimates based on directly observed PGSI responses with alternative representations of structurally non-applicable responses. The broader PGSI ≥3 threshold was also examined to determine whether descriptive conclusions depended on the conventional ≥8 threshold. Multivariable regression analyses used complete-case analysis based on the variables included in each model. Statistical significance was set at *p* < 0.05. Primary statistical analyses were performed using IBM SPSS Statistics Version 29.0 (IBM Corp., Armonk, NY, USA). Additional bootstrap, post-hoc, and model-performance analyses were performed using Python, Version 3.14.7 (Python Software Foundation, Wilmington, DE, USA), with SciPy, Version 1.18.0 (SciPy Developers), statsmodels, Version 0.14.6 (statsmodels Developers), and scikit-learn, Version 1.9.0 (scikit-learn Developers).

### 3.6. Ethics Statement

The study was conducted in accordance with the Declaration of Helsinki, and approved by the Ethics Committee of Zhetysu University (Protocol No. 2025-04/K, 7 April 2025). Informed consent was obtained from all subjects involved in the study. Participation was voluntary and respondents were informed of their right to withdraw at any stage without consequence. Personally identifiable information was not collected or retained.

## 4. Results

### 4.1. Psychometric Properties of the Bilingual PGSI

Among the 474 respondents with directly observed responses across all nine PGSI items, internal consistency was good (Cronbach’s α = 0.846). Corrected item–total correlations ranged from 0.359 to 0.693. Sampling adequacy was good (KMO = 0.881), and Bartlett’s test was significant, χ^2^(36) = 1465.46, *p* < 0.001. The first principal component accounted for 45.9% of the total variance, with item loadings ranging from 0.439 to 0.792.

CFA conducted in the same directly assessed subgroup (n = 474) supported a dominant one-factor structure, χ^2^(27) = 119.69, *p* < 0.001, CFI = 0.985, TLI = 0.981, RMSEA = 0.085 (95% CI [0.067, 0.104]), and SRMR = 0.069. Standardized factor loadings ranged from 0.448 to 0.835. Although CFI and TLI indicated favorable comparative fit, RMSEA suggested some residual absolute misfit; accordingly, the findings support a dominant common dimension rather than unequivocal strict unidimensionality (Table 2).

### 4.2. Sample Characteristics and Distribution of PGSI Categories

Among the 474 respondents who directly completed the PGSI (Table 3), the mean score was 8.56 (SD = 5.03), median = 9 (IQR = 5–12), with a range of 0–24. Thirty-three respondents (7.0%) scored 0, 30 (6.3%) scored 1–2, 136 (28.7%) scored 3–7, and 275 (58.0%) scored ≥ 8. The same 275 respondents represented 27.1% of the overall survey sample (275/1015); these percentages therefore reflect different denominator).

Independent reconstruction of PGSI total scores from the original item-level responses reproduced the classification exactly, with 275 of 474 directly assessed respondents (58.0%) scoring ≥ 8. The same 275 respondents constituted 27.1% of the overall survey sample (275/1015). Thus, the two percentages reflect different analytic denominators rather than different numbers of classified individuals. Across individual PGSI items, “Sometimes” was generally the most frequently endorsed non-zero response category (Table 4). The distribution of PGSI categories in the directly assessed and overall survey samples is presented in Table 5.

PGSI ≥ 8 classification did not differ significantly by age group, χ^2^(3) = 2.43, *p* = 0.488, student status, χ^2^(1) = 0.04, *p* = 0.837, or broad geographic grouping, χ^2^(1) = 3.19, *p* = 0.074. In contrast, PGSI ≥ 8 was more frequent among respondents reporting regular gambling (141/188, 75.0%) than among those reporting rare or episodic gambling (134/286, 46.9%), χ^2^(1) = 35.75, *p* < 0.001.

Sensitivity analyses confirmed that the treatment of structurally non-applicable responses materially affected psychometric estimates. Cronbach’s α was 0.846 in the directly assessed subgroup (n = 474), 0.937 when respondents explicitly reporting no past-year gambling were represented as structural zeros while the 12 uncertain screening responses were excluded (n = 1003), and 0.938 in the previous full-sample representation (N = 1015). Corresponding first-component variance estimates were 45.9%, 67.1%, and 67.3%, respectively. The number of respondents meeting PGSI ≥ 8 remained unchanged (n = 275). Using the broader PGSI ≥ 3 threshold, 411 of 474 directly assessed respondents (86.7%) met the threshold.

### 4.3. Distribution of Gambling-Related Harms

Item-level analyses demonstrated variability in endorsement frequencies across the nine PGSI indicators (Table 4). For each item, the largest proportion of positive responses was observed within the response category “Sometimes”, whereas endorsement of “Most of the time” and particularly “Almost always” was substantially less frequent.

The highest frequencies of positive responses were observed for behavioral indicators reflecting repeated gambling involvement, whereas financial and interpersonal consequences were endorsed less frequently but remained consistently represented across the study population. Responses indicating the highest severity (“Almost always”) accounted for only a small proportion of endorsements across all PGSI items.

### 4.4. Bivariate Associations with PGSI ≥ 8 Screening Status

Bivariate analyses identified statistically significant associations between problem gambling (PGSI ≥ 8) and several demographic and gambling-related characteristics (Table 6).

Sex was significantly associated with problem gambling (χ^2^ = 23.20, df = 1, *p* < 0.001, Cramér’s V = 0.151). Occupational status also demonstrated a statistically significant association (χ^2^ = 29.88, df = 13, *p* = 0.005, Cramér’s V = 0.172). In contrast, neither age group (χ^2^ = 6.30, df = 3, *p* = 0.098) nor marital status (χ^2^ = 7.29, df = 6, *p* = 0.295) was significantly associated with PGSI ≥ 8.

Among gambling-related variables, the strongest associations were observed for gambling frequency (χ^2^ = 91.61, df = 4, *p* < 0.001, Cramér’s V = 0.440) and the proportion of monthly income spent on gambling (χ^2^ = 86.57, df = 3, *p* < 0.001, Cramér’s V = 0.427). Duration of gambling involvement was also significantly associated with problem gambling (χ^2^ = 13.53, df = 4, *p* = 0.009), although the corresponding effect size was comparatively small (Cramér’s V = 0.169).

The significant omnibus association between occupation and PGSI ≥ 8 status (χ^2^(13) = 29.88, *p* = 0.005, Cramér’s V = 0.172) was further examined using adjusted standardized residuals. The association was driven primarily by respondents employed in industry/construction, among whom 39.1% (43/110) exceeded the PGSI ≥ 8 threshold; this subgroup showed the largest positive adjusted residual (z = 3.00). Thus, the occupational association did not reflect a uniform difference across all occupational categories.

### 4.5. Multivariable Logistic Regression

A multivariable logistic regression model was constructed to identify correlates of PGSI ≥ 8 screening status (Table 7).

After simultaneous adjustment for all variables included in the model, the proportion of monthly income spent on gambling showed the strongest association with PGSI ≥ 8 screening status (adjusted OR = 2.80, 95% CI: 1.90–4.12, *p* < 0.001). Gambling frequency was also significantly associated with PGSI ≥ 8 screening status (adjusted OR = 2.13, 95% CI: 1.62–2.80, *p* < 0.001). By contrast, sex was no longer significantly associated with problem gambling after adjustment (adjusted OR = 1.16, 95% CI: 0.70–1.90, *p* = 0.565). Likewise, none of the age categories was significantly associated with PGSI ≥ 8 screening status after adjustment. Duration of gambling involvement also failed to retain statistical significance in the adjusted model (adjusted OR = 1.14, 95% CI: 0.93–1.41, *p* = 0.212). Overall, gambling-related behavioral and financial variables showed stronger adjusted associations with PGSI ≥ 8 screening status than the demographic variables examined.

Age was not significantly associated with PGSI ≥ 8 status when evaluated jointly across all age categories in the multivariable model (likelihood-ratio χ^2^(3) = 2.48, *p* = 0.478). The model demonstrated moderate explanatory capacity (Nagelkerke R^2^ = 0.310) and good discrimination (AUC = 0.800). However, the Hosmer–Lemeshow test was statistically significant, χ^2^(8) = 19.83, *p* = 0.011, indicating some evidence of imperfect calibration; accordingly, the model’s predicted probabilities should be interpreted cautiously.

### 4.6. Continuous PGSI Severity Analysis

To examine whether the observed associations were dependent on dichotomization at the PGSI ≥ 8 threshold, an additional multiple linear regression was conducted using the total PGSI score as a continuous outcome. The results were broadly consistent with the primary binary logistic model (Table 8). Greater gambling expenditure was associated with higher PGSI scores (B = 2.63, 95% CI 2.05–3.20, *p* < 0.001), as was greater gambling frequency (B = 1.58, 95% CI 1.16–2.00, *p* < 0.001). Gambling duration showed a smaller positive association (B = 0.44, 95% CI 0.06–0.81, *p* = 0.023), whereas sex and most age categories were not statistically significant. The model explained 41.2% of the variance in continuous PGSI scores (R^2^ = 0.412; adjusted R^2^ = 0.403).

### 4.7. Sensitivity Analysis of Ordinal Predictor Specification

Sensitivity analyses provided no statistically significant evidence of departure from a linear trend for the share of income spent on gambling, χ^2^(2) = 5.56, *p* = 0.062. In contrast, the categorical specification provided a better fit for gambling frequency, χ^2^(3) = 14.09, *p* = 0.003, indicating that its association with PGSI ≥ 8 status was not adequately characterized by a constant per-category effect. Accordingly, the corresponding per-category OR reported in Table 7 should be interpreted as a summary trend across the ordered frequency categories rather than as an identical change in odds between adjacent categories.

### 4.8. Cross-Language Psychometric Performance and Measurement Invariance

Language-stratified analyses indicated high internal consistency for both language versions. Cronbach’s α was 0.938 for the Russian-language version (n = 527) and 0.937 for the Kazakh-language version (n = 488), compared with 0.938 in the combined sample (N = 1015) (Table 3).

Multi-group CFA was subsequently used to examine measurement invariance across the Russian- and Kazakh-language versions. The configural model yielded CFI = 0.927, TLI = 0.903, RMSEA = 0.097, and SRMR = 0.051. Imposing equality constraints on factor loadings resulted in minimal deterioration in comparative fit (metric model: ΔCFI = −0.001; ΔRMSEA = −0.006). Additional scalar constraints likewise produced only small changes (ΔCFI = −0.001; ΔRMSEA = −0.004). Although absolute model fit was not uniformly optimal, particularly for RMSEA, the small changes in comparative fit indices across increasingly constrained models provided preliminary support for metric and scalar measurement invariance between the Kazakh- and Russian-language versions.

## 5. Discussion

### 5.1. Psychometric Performance of the Bilingual PGSI

Among respondents who directly completed the PGSI, the instrument demonstrated good internal consistency (α = 0.846), good sampling adequacy (KMO = 0.881), and a dominant first component accounting for 45.9% of the variance. The substantially higher estimates obtained under structural-zero coding demonstrate that structurally non-applicable responses materially influence covariance-based psychometric indices. Primary psychometric interpretation is therefore based on directly observed PGSI responses. CFA provided further support for a dominant common PGSI dimension, with favorable CFI and TLI values and acceptable residual fit. However, the elevated RMSEA indicated some remaining absolute model misfit. Accordingly, the findings support a dominant underlying severity dimension (Miller et al., 2013; Sharp et al., 2012; López-González et al., 2018a), but should not be interpreted as unequivocal evidence of strict unidimensionality.

Cross-language analyses indicated broadly similar measurement structure across the Russian- and Kazakh-language versions, but complete measurement invariance was not established. Configural fit was acceptable, whereas equality constraints on factor loadings produced a meaningful deterioration in fit. Additional threshold constraints produced comparatively little further deterioration. These findings are broadly consistent with evidence that the PGSI can retain satisfactory psychometric properties across linguistic and cultural adaptations (Sharp et al., 2012; Bertossa et al., 2014; López-González et al., 2018b), while also supporting recommendations that measurement equivalence should be empirically evaluated rather than assumed (International Test Commission [ITC], 2018). Accordingly, the present findings should be interpreted as preliminary evidence of cross-language psychometric comparability rather than as definitive evidence of measurement equivalence.

### 5.2. Response Pattern Across PGSI Items

One of the principal findings of this study was that 58.0% of respondents who directly completed the PGSI (275/474) met the PGSI ≥ 8 screening threshold; the same 275 respondents represented 27.1% of the overall survey sample (275/1015). This comparatively high proportion should be interpreted cautiously in light of the demographic and methodological characteristics of the study. Younger adults and students were prominent in the sample, while mixed online–offline recruitment may have introduced some degree of self-selection. Importantly, item-level distributions showed that “Sometimes” responses were substantially more frequent than “Almost always” responses. Given the additive structure of the PGSI, accumulation of moderate-frequency endorsements across several domains can result in scores ≥ 8 without consistently severe endorsement of individual items, consistent with evidence that PGSI items represent different locations along a continuum of gambling-problem severity (Miller et al., 2013; Sharp et al., 2012). Cultural and linguistic variation in the interpretation of items concerning guilt, social criticism, affordability, and financial consequences also cannot be excluded, as score equivalence cannot be assumed from translation alone (Van de Vijver & Poortinga, 1997; International Test Commission [ITC], 2018). Accordingly, the observed 27.1% should be interpreted as the proportion exceeding the PGSI screening threshold in this multi-regional sample rather than as a national estimate of clinically diagnosed gambling disorder.

This finding should also be considered within the broader national context. Official administrative data indicate substantial gambling involvement and demand for voluntary self-exclusion in Kazakhstan. According to the Ministry of Tourism and Sports of the Republic of Kazakhstan (2024b), approximately 350,000 citizens were estimated to participate in gambling and sports betting, while approximately 130,000 individuals had voluntarily restricted their participation through the eGov Mobile self-exclusion service following its introduction in March 2024. The national Comprehensive Plan for 2024–2026 subsequently established a policy target to reduce the number of citizens regularly participating in gambling and betting from approximately 350,000 to 200,000 (Ministry of Tourism and Sports of the Republic of Kazakhstan, 2024a). These administrative indicators are not epidemiologically equivalent to PGSI-defined problem gambling and therefore cannot validate the high PGSI ≥ 8 proportion observed in the present sample. Nevertheless, they provide independent contextual evidence that the observed distribution occurred within a setting characterized by substantial documented gambling involvement and demand for self-exclusion.

### 5.3. Behavioral and Demographic Correlates of PGSI-Defined Severity

In the multivariable model, gambling-related behavioral characteristics showed stronger associations with PGSI ≥ 8 status than the demographic characteristics examined. Gambling expenditure and frequency showed the strongest adjusted associations, whereas sex, age, and gambling duration were not statistically significant after adjustment. However, the precision of several demographic estimates was limited by comparatively wide confidence intervals, precluding strong conclusions regarding the absence of demographic associations. Moreover, expenditure and frequency are conceptually proximal to dimensions represented within the PGSI, creating potential criterion contamination despite the absence of problematic statistical multicollinearity. These variables should therefore be interpreted as behavioral correlates of PGSI-defined severity rather than independent causal determinants (Dellis et al., 2014; Miller et al., 2013).

Importantly, the additional analysis treating the total PGSI score as a continuous outcome yielded a broadly consistent pattern, with gambling expenditure and frequency remaining positively associated with PGSI severity. Gambling duration also showed a smaller positive association in the continuous model. This convergence across alternative outcome specifications suggests that the principal behavioral associations were not solely attributable to dichotomization at the PGSI ≥ 8 threshold. More broadly, these findings support interpretation of PGSI-defined severity along a continuum rather than as a sharp distinction between individuals immediately below and above the conventional screening threshold. This interpretation is consistent with recent research using PGSI scores as a continuous measure of gambling-problem severity and with contemporary psychometric evidence supporting a general severity dimension underlying PGSI responses (Tabri & Wohl, 2023). Accordingly, the logistic model is presented as an analysis of sample-specific behavioral correlates rather than as a calibrated individual-level prediction instrument.

### 5.4. Public Health and Practical Implications

From a public-health perspective, the present findings support consideration of brief gambling-harm screening within healthcare and community settings in Kazakhstan, particularly primary care and mental-health/addiction services. Recent evidence indicates that integrating gambling-harm screening into general practice and community services is feasible, while also highlighting the importance of appropriate staff training and clearly defined referral pathways (Reid et al., 2024). More broadly, contemporary public-health evidence supports early identification of gambling-related harm within healthcare systems while emphasizing that screening should form part of a wider prevention and care pathway rather than function as a stand-alone intervention (Clune et al., 2024; LaPlante & Gray, 2026). In Kazakhstan, systematic collection of such screening data could additionally provide a basis for monitoring gambling-related harm and informing locally adapted prevention strategies. Accordingly, positive screening results should prompt further clinical assessment rather than be interpreted as diagnostic classifications.

From a computational social science perspective, the PGSI ≥ 8 cutoff can be conceptualized as an administrative boundary imposed on a continuous spectrum of gambling-related risk. In this interpretation, the threshold functions as an operational indicator for health monitoring and resource allocation rather than as a categorical definition of an individual’s identity or social status. Crossing the threshold may therefore serve as a trigger for the system to initiate further assessment or offer appropriate support, such as counseling, rather than to assign a fixed label to the individual. This distinction is particularly important when risk scores are incorporated into digital or administrative systems, where algorithmic classifications may influence eligibility for care or allocation of services and may be limited by incomplete information about individuals’ social and clinical circumstances (Nong & Adler-Milstein, 2021). Conceptualizing the PGSI ≥ 8 cutoff in this way preserves its practical function as an informational decision point while reducing the risk that a screening classification is transformed into a stigmatizing or discriminatory social label.

To translate this principle into practice, screening-based identification should be accompanied by proportionate and non-stigmatizing support mechanisms that preserve individuals’ social and economic participation. In workplace settings, these may include confidential employee assistance or referral pathways that facilitate access to counseling without automatically linking help-seeking or screening results to employment decisions. In healthcare and community settings, anonymous or low-threshold early-intervention options may reduce barriers to seeking help at an earlier stage. Financial institutions may also provide voluntary, user-controlled safeguards, such as self-imposed spending or transaction limits and other mechanisms that allow individuals to restrict gambling-related access (World Health Organization, 2024). Such approaches can be conceptualized as forms of reasonable accommodation: rather than excluding individuals on the basis of a screening classification, they use the information generated by screening to facilitate proportionate support and harm reduction. The effectiveness, acceptability, and appropriate implementation of these measures should be evaluated in future research and adapted to the Kazakhstani legal, healthcare, workplace, and financial contexts.

In the Kazakhstani context, however, the translation of screening into specific public-health or policy interventions should be viewed as a sequential process. A necessary first step is to establish a culturally and linguistically validated instrument capable of reliably identifying gambling-related risk in both Kazakh- and Russian-speaking populations. The present study addresses this foundational stage. Subsequent research should determine where and how screening can be feasibly implemented, establish appropriate referral pathways, and evaluate the acceptability and effectiveness of specific support measures before broader policy recommendations are made.

These findings are also relevant to the United Nations 2030 Agenda for Sustainable Development, particularly Sustainable Development Goal 3 (Good Health and Well-being) and Target 3.4, which aims to reduce premature mortality from non-communicable diseases and promote mental health and well-being by 2030 (United Nations, 2015). In this context, the availability of a culturally and linguistically appropriate screening instrument may facilitate the earlier identification of gambling-related harm and inform more responsive public health strategies in Kazakhstan.

### 5.5. Limitations

Several limitations should be considered. Only 474 respondents provided directly observed responses across all nine PGSI items. Primary psychometric conclusions are therefore based on this subgroup. Respondent-level recruitment-mode identifiers were not retained in the final analytic dataset, preventing reliable retrospective comparison of online and offline recruitment channels. The unusually high PGSI ≥ 8 proportion further underscores the need for independent replication and external criterion validation.

Although the sample covered all 17 regions and three cities of republican significance in Kazakhstan, its comparatively young age structure, substantial student representation, and mixed online–offline recruitment may limit population representativeness and introduce self-selection; institutionalized populations were also not included. Accordingly, the proportion exceeding the PGSI ≥ 8 threshold should not be interpreted as a national prevalence estimate. Although cross-language analyses provided preliminary evidence of comparability between the Russian- and Kazakh-language versions, culturally or linguistically patterned differences in item interpretation cannot be entirely excluded. The factorial structure was evaluated within the same sample without independent or split-sample cross-validation, and test–retest reliability and convergent/discriminant validity against independent measures were not assessed. Finally, the cross-sectional, self-report design precludes causal inference and remains susceptible to recall and social-desirability biases. Future studies should address these limitations using probability-based sampling, independent validation samples, and longitudinal and convergent validation designs. Future research should also examine how screening-defined gambling risk may inform effective and non-stigmatizing support strategies across healthcare, workplace, and financial-service settings.

## 6. Conclusions

This study provides the first comprehensive evaluation of the bilingual Russian–Kazakh version of the Problem Gambling Severity Index (PGSI) in a large multi-regional sample from Kazakhstan. The findings support the internal consistency of the PGSI and a dominant common severity dimension among respondents who directly completed the instrument. However, the elevated screening-score distribution, non-probability sampling design, and absence of external criterion validation require cautious interpretation. Further independent validation is needed before population-level screening applications can be recommended.

A substantial proportion of participants in the present study sample met the conventional PGSI screening threshold of ≥8 for the highest-risk/problem-gambling category. This proportion should be interpreted as a sample-specific finding and should not be considered a nationally representative estimate. Item-level analyses further showed that elevated PGSI scores were more commonly characterized by the accumulation of moderate gambling-related problems across multiple domains than by frequent endorsement of the most severe response category. In addition, behavioral indicators—particularly gambling expenditure and gambling frequency—showed the strongest adjusted associations with PGSI-defined severity.

These findings extend the evidence on the PGSI in culturally diverse populations and provide the first psychometric evidence for its application in Kazakhstan. They also highlight the value of behavioral and financial indicators for the early identification of gambling-related harm and provide an empirical basis for future epidemiological research, prevention strategies, and public health initiatives addressing gambling-related harm in Kazakhstan.

## Figures and Tables

**Table 1:** Sociodemographic characteristics of the study sample (valid %).

Variable	Category	n	%
Gender	Male	551	54.3
	Female	464	45.7
Age group	18–21	302	29.8
	22–35	306	30.1
	36–45	301	29.7
	46–60	106	10.4
Marital status	Married	498	49.1
	Single	434	42.8
	Divorced	52	5.1
	Widowed	9	0.9
	Other	22	2.2
Occupation	Student	205	20.2
	Trade/Services	165	16.3
	Industry/Construction	110	10.8
	Education/Science	101	10.0
	Business/Management	75	7.4
	Transport/Logistics	66	6.5
	Unemployed	64	6.3
	Other occupations *	229	22.5
Income spent on gambling	<10%	287	28.3
	10–25%	128	12.6
	26–50%	42	4.1
	>50%	17	1.7

* Other occupations include all occupational categories not listed above.

**Table 2:** Psychometric properties and factorial structure of the PGSI.

**A. Overall psychometric and CFA fit indices**		
**Psychometric indicator**	**Value**	
Cronbach’s α	0.846	
KMO	0.881	
Bartlett’s test, χ^2^(36)	1465.46	
*p*	<0.001	
PCA: variance explained by first component	45.9%	
CFA: χ^2^(27)	119.69	
CFA: CFI	0.985	
CFA: TLI	0.981	
CFA: RMSEA (95% CI)	0.085 [0.067, 0.104]	
CFA: SRMR	0.069	
Standardized CFA loading range	0.448–0.835	
**B. Standardized CFA factor loadings**		
**PGSI item**	**Item–total r**	**PCA loading**
Bet more than could afford	0.608	0.724
Needed larger amounts	0.560	0.684
Chasing losses	0.593	0.715
Borrowed/sold to gamble	0.633	0.750
Felt might have a problem	0.424	0.510
Health problems/stress	0.591	0.689
Others criticized gambling	0.617	0.717
Financial problems	0.693	0.792
Guilt	0.359	0.439

Note: α = 0.846; KMO = 0.881; Bartlett χ^2^(36) = 1465.46, *p* < 0.001; first component = 45.9%.

**Table 3:** Language-stratified reliability and cross-language measurement invariance of the PGSI.

**A. Internal consistency**							
**Language version**		**n**				**Cronbach’s α**	
Russian		255				0.864	
Kazakh		219				0.820	
**B. Multi-group ordinal CFA measurement invariance**							
**Model**	**χ^2^**	**df**	**CFI**	**TLI**	**RMSEA (95% CI)**	**ΔCFI**	**ΔRMSEA**
Configural	166.01	54	0.984	0.978	0.066 [0.053, 0.080]	−	−
Loading invariance	275.08	63	0.969	0.964	0.084 [0.072, 0.097]	−0.015	+0.018
Threshold invariance	340.36	90	0.963	0.971	0.077 [0.067, 0.087]	−0.006	−0.007

Note: Analyses were conducted among respondents with directly observed responses to all nine PGSI items (n = 474; Russian-language version, n = 255; Kazakh-language version, n = 219).

**Table 4:** Distribution of response categories across individual PGSI items among directly assessed respondents (n = 474).

PGSI Item	Never n (%)	Sometimes n (%)	Most of the Time n (%)	Almost Always n (%)
Bet more than could afford	120 (25.3)	259 (54.6)	78 (16.5)	17 (3.6)
Needed larger amounts	133 (28.1)	253 (53.4)	69 (14.6)	19 (4.0)
Chasing losses	83 (17.5)	230 (48.5)	100 (21.1)	61 (12.9)
Borrowed/sold to gamble	181 (38.2)	214 (45.1)	60 (12.7)	19 (4.0)
Felt might have a problem	188 (39.7)	202 (42.6)	60 (12.7)	24 (5.1)
Health problems/stress	211 (44.5)	181 (38.2)	63 (13.3)	19 (4.0)
Others criticized gambling	139 (29.3)	211 (44.5)	90 (19.0)	34 (7.2)
Financial problems	182 (38.4)	192 (40.5)	74 (15.6)	26 (5.5)
Guilt	145 (30.6)	224 (47.3)	67 (14.1)	38 (8.0)

Note: Percentages are calculated among the 474 respondents with directly observed responses to all nine PGSI items. Structurally non-applicable responses from participants who were not administered the PGSI are not included in these item-level distributions.

**Table 5:** Distribution of PGSI categories in the study sample.

PGSI Category	Directly Assessed n = 474	Overall Survey N = 1015
0	33 (7.0%)	574 (56.6%)
1–2	30 (6.3%)	30 (3.0%)
3–7	136 (28.7%)	136 (13.4%)
≥8	275 (58.0%)	275 (27.1%)

**Table 6:** Associations of demographic and gambling-related characteristics with PGSI ≥ 8 screening status.

Predictor	χ^2^ (df)	*p*-Value	Cramér’s V	95% CI
Sex	23.20 (1)	<0.001	0.151	0.096–0.205
Age group	6.30 (3)	0.098	0.079	0.038–0.149
Marital status	7.29 (6)	0.295	0.085	0.056–0.162
Occupation	29.88 (13)	0.005	0.172	0.151–0.251
Share of monthly income spent on gambling	86.57 (3)	<0.001	0.427	0.353–0.504
Gambling frequency	91.61 (4)	<0.001	0.440	0.373–0.516
Duration of gambling involvement	13.53 (4)	0.009	0.169	0.102–0.273

Note: V = Cramér’s V. 95% CIs for Cramér’s V were obtained using nonparametric bootstrap resampling (3000 resamples).

**Table 7:** Multivariable logistic regression predicting PGSI ≥ 8 screening status.

Predictor	Adjusted OR	95% CI	*p*-Value
Male sex (vs. female)	1.16	0.70–1.90	0.565
Age 22–35 years	0.65	0.38–1.13	0.126
Age 36–45 years	0.79	0.44–1.42	0.430
Age 46–60 years	0.69	0.31–1.52	0.354
Share of income spent on gambling (per category increase)	2.80	1.90–4.12	<0.001
Gambling frequency (per category increase)	2.13	1.62–2.80	<0.001
Duration of gambling involvement (per category increase)	1.14	0.93–1.41	0.212

Note: OR = odds ratio; CI = confidence interval. Reference categories were female sex and age 18–21 years. Multicollinearity diagnostics indicated no evidence of problematic collinearity among predictors, with VIF values ranging from 1.04 to 1.57; the VIFs for the share of income spent on gambling and gambling frequency were 1.31 and 1.29, respectively.

**Table 8:** Multiple linear regression predicting continuous PGSI total score.

Predictor	B	95% CI	*p*-Value
Male sex (vs. female)	−0.57	−1.41–0.28	0.187
Age 22–35 years	−1.00	−1.90–−0.11	0.027
Age 36–45 years	−0.71	−1.72–0.30	0.169
Age 46–60 years	−1.13	−2.53–0.28	0.116
Share of income spent on gambling (per category increase)	2.63	2.05–3.20	<0.001
Gambling frequency (per category increase)	1.58	1.16–2.00	<0.001
Duration of gambling involvement (per category increase)	0.44	0.06–0.81	0.023

Note: B = unstandardized regression coefficient; CI = confidence interval. Reference categories were female sex and age 18–21 years. Higher values for gambling frequency and share of income spent on gambling represent greater gambling frequency and a larger share of income spent, respectively. HC3 heteroscedasticity-robust standard errors were used. Overall model performance: R^2^ = 0.412; adjusted R^2^ = 0.403; F(7, 466) = 37.40, *p* < 0.001.

## Data Availability

Data available in a publicly accessible repository. The original data presented in the study are openly available in Zenodo at https://doi.org/10.5281/zenodo.21717164.

## Abbreviations

The following abbreviations are used in this manuscript:

PGSI	Problem Gambling Severity Index
DSM	Diagnostic and Statistical Manual of Mental Disorders
ICD	International Classification of Diseases
